# ERO1α as a Potential Drug Target for Breast Cancer: A Systematic Review of Current Evidence

**DOI:** 10.3390/ijms262110276

**Published:** 2025-10-22

**Authors:** Kamilla Khojayeva, Aiman Moldasheva, Mohamad Aljofan

**Affiliations:** 1 Department of Biomedical Sciences, School of Medicine Nazarbayev University, Astana 010000, Kazakhstan; kamilla.khojayeva@nu.edu.kz (K.K.); aiman.moldasheva@nu.edu.kz (A.M.); 2 Drug Discovery and Development, Center of Life Sciences, National Laboratory, Astana 010000, Kazakhstan

**Keywords:** ERO1α, breast cancer, oxidative stress, drug target

## Abstract

Hypoxia, oxidative stress, and impaired protein folding contribute to tumor progression and therapy resistance. Endoplasmic Reticulum Oxidoreductin 1 Alpha (ERO1α) is a key enzyme regulating redox homeostasis in the endoplasmic reticulum by reoxidizing protein disulfide isomerase, facilitating disulfide bond formation, and generating reactive oxygen species. Elevated ERO1α levels are associated with increased tumor aggressiveness, metastasis, and poor clinical outcomes. Despite growing evidence of its tumor-promoting functions, no clinically approved ERO1α inhibitors exist. This systematic review provides a comprehensive and integrative analysis of current research on ERO1α in breast cancer, emphasizing its roles in hypoxia response, angiogenesis, immune modulation, and ferroptosis resistance. We discuss mechanistic links, including VEGF-A maturation and PD-L1-mediated immune evasion, and highlight recent advances in small-molecule ERO1α inhibitors and preclinical therapeutic strategies. By consolidating molecular insights and translational considerations, this review underscores ERO1α as both a promising therapeutic target and potential prognostic marker, offering guidance for future drug development and targeted interventions in redox-dependent cancer pathways.

## 1. Introduction

Tumor progression in breast cancer is driven by complex mechanisms involving genetic mutations, dysregulated signaling pathways [1] and the tumor microenvironment [2]. Key factors such as oxidative stress [3] and hypoxia [4] play significant roles in breast cancer progression, influencing cell survival and resistance to therapy. Oxidative stress, a consequence of an imbalance between ROS production and antioxidant defense mechanisms, is a hallmark of cancer biology, contributing to tumorigenesis, metastasis, and therapy resistance [5]. Notably, within the endoplasmic reticulum (ER), which is responsible for the proper folding of proteins, oxidative stress is a direct result of the enzymatic activity required to maintain cellular homeostasis during protein synthesis [6].

The ER plays a central role in protein folding and cellular homeostasis, and its stress response is closely linked to cancer survival. A key contributor to oxidative folding in the ER is Endoplasmic Reticulum Oxidoreductin 1 alpha (ERO1α), which transfers electrons to protein disulfide isomerase (PDI) during disulfide bond formation, maintaining proper protein structure and function [7,8]. By reoxidizing PDI, ERO1α also generates reactive oxygen species (ROS). Tu and Weissman (2002) [9] estimated that ERO1-mediated oxidation could account for up to ~25% of cellular ROS during protein synthesis, based on biochemical assumptions about ATP usage, secretory protein proportion, and ROS generation. Although theoretical and cell-type dependent, this highlights the substantial oxidative burden of disulfide bond formation. Supporting this, Princiotta et al. showed that protein synthesis is a major driver of cellular energy consumption, further emphasizing the metabolic significance of ERO1 activity [10].

Recently, ERO1α has gained attention for its upregulation in several cancer types, particularly in hypoxic and highly secretory tumor environments. Multiple studies have demonstrated its overexpression in aggressive cancers such as pancreatic [11], hepatic [12], breast [13], and lung cancer [14], associating it with poor prognosis, increased invasiveness, and therapy resistance [15]. ERO1α plays a crucial role in maintaining redox balance within the endoplasmic reticulum, yet its dysregulation in cancer has been associated with increased oxidative stress and alterations in cellular metabolism. Elevated ERO1α expression may contribute to tumor adaptation by influencing protein folding efficiency, modulating stress response pathways, and promoting interactions between cancer cells and their microenvironment [16].

Despite the growing interest in ERO1α and its function in cancer development, there is a lack of comprehensive literature on its expression and impact on various breast cancer subtypes and its correlation with clinical outcomes. The lack of studies that evaluate and systematically analyze the available ERO1α results have partly contributed to the ambiguity around ERO1α, and whether it could be used as a reliable marker to predict the outcome of a cancer diagnosis or an effective target for treatment.

Therefore, this study seeks to fill the gap by providing a summary of ERO1α expression patterns and investigating the important molecular pathways where ERO1α overexpression is involved, including oxidative stress, hypoxia response, immunological modulation, and unfolded protein response. While there are no medicines that specifically target ERO1α, this systematic review’s findings should direct future drug development efforts by drawing attention to the significance of ERO1α overexpression in breast cancer and its role in important pathways that promote tumor growth.

## 2. Methods

The present systematic review was performed according to the guidelines of “The PRISMA 2020 statement: an updated guideline for reporting systematic reviews” by Page et al., 2021 [17]. As required by the guidelines for methodologically sound drafting of systematic reviews, the protocol of this systematic review was registered with Open Science Framework [18].

Search strategy:

Three electronic databases (PubMed, Scopus, ScienceDirect) were searched for articles related to the ERO1α and its role in breast cancer development and progression. As this topic is relatively new, we did not limit the start date for the publication, but the end date was established to be 31 May 2025. The following key words were used for the search: (“ERO1α” OR “ERO1 alpha” OR “Endoplasmic reticulum oxidoreductin 1 alpha” OR “ERO1L” OR “ERO1Lα” OR “Ero1alpha”) AND (“breast cancer” OR “breast tumor” OR “mammary carcinoma”).

Eligibility criteria:

For articles selection several inclusion and exclusion criteria were established.

Inclusion criteria are as follows:

- Primary research articles focused on ERO1α in breast cancer.
- In vitro and in vivo studies.
- Articles published in English.

Exclusion criteria are following:

- Articles published in languages other than English.
- Reviews and meta-analyses.
- Studies not related to ERO1α or breast cancer.

Data collection process:

K Khojayeva and A Moldasheva independently conducted the study search, selection, and quality assessment. The selected list of articles was compared. Disagreements were solved by discussion with M Aljofan. The quality of the studies included was assessed according to the quality of the body of evidence in the GRADE approach that are applicable to in vitro studies, including risk of bias, inconsistency, and imprecision. The assessment was conducted in two stages: in the first stage, we evaluated the titles and performed abstract screening, and in the second stage, we thoroughly conducted full-text assessment of the remaining articles to ensure that they meet eligibility criteria.

Types of outcome:

The primary outcome of interest is the mechanistic role of ERO1α in breast cancer development and progression.

Data extraction:

The extracted data included the name of the first author; publication year; methodology; and key results.

## 3. Results

**Figure 1:**
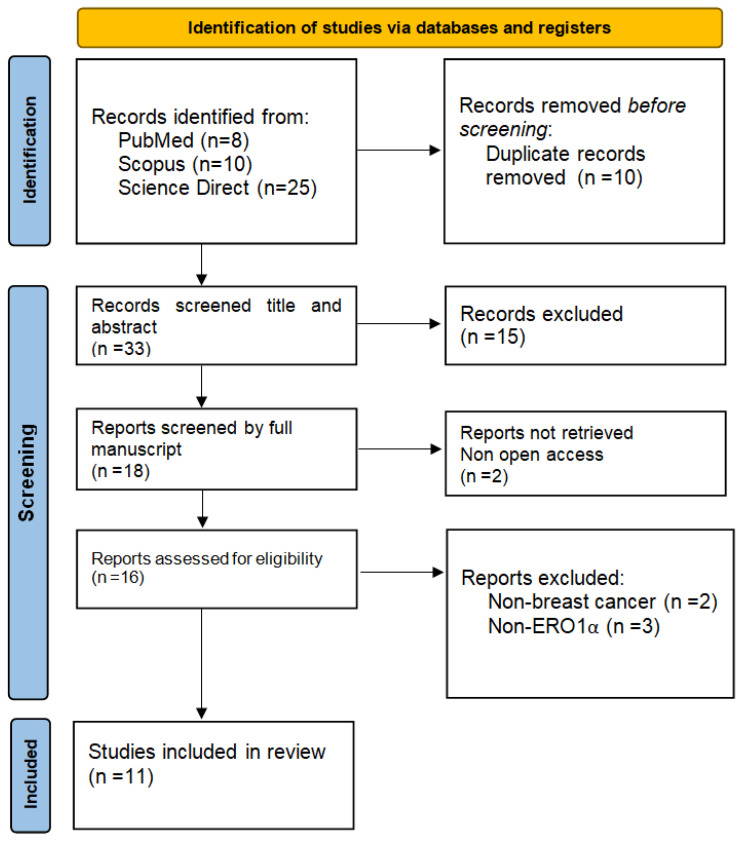
Prisma flow-chart showing the search strategy. Initial search included 43 articles. After abstract screening and full-text review, 11 original articles were included in the systematic review.

A total of 43 records were identified across the three databases. After removing 10 duplicate entries, 33 records remained for screening. Following title and abstract evaluation, 15 articles were excluded as irrelevant because they either did not investigate ERO1α or did not address breast cancer. Full-text assessment was performed for the remaining 18 articles, of which 7 were excluded for the following reasons: 2 articles could not be retrieved despite attempts to access the full text, 3 did not focus on breast cancer (e.g., studies on cervical cancer [19], atherosclerosis [20] or cholangiocarcinoma [21]), and 2 did not directly evaluate ERO1α in the context of breast cancer progression (e.g., a docking study of inhibitors [22] or macrophage-induced invasion of breast epithelial cells without direct analysis of breast tumors [23]). Ultimately, 11 studies met the eligibility criteria and were included in the final analysis (Figure 1). A summary of the key findings from these 11 studies is provided in Table 1.

### 3.1. Overexpression of ERO1α in Breast Cancer Cells Compared to Normal Cells

Recently ERO1α has gained increased attention for its role in cancer progression. High levels of this protein are often linked to more aggressive tumors, increased metastasis, and worse patient outcomes. Studies show that ERO1α is overexpressed in breast cancer cell lines and tissues. In contrast, it is either low or absent in normal mammary gland tissue.

Kutomi et al. (2013) was among the first to report high ERO1α expression in the MCF-7 breast cancer cell line [24]. Out of 71 normal breast samples, none showed ERO1α staining. Additionally, ERO1α mRNA was also detected in breast cancer tissues but not in healthy mammary glands.

Tanaka et al. (2015) expanded on these findings [25]. Authors confirmed that ERO1α expression was significantly higher in breast cancer cell lines and tissues than in normal breast tissue. This upregulation occurred across different breast cancer subtypes. Their immunohistochemical analysis showed that tumor cells expressed ERO1α, while normal tissues did not. They also observed patchy expression within tumors, possibly due to variations in oxygen availability. This further supports the idea that hypoxia plays a role in regulating ERO1α levels.

In their follow up study, Tanaka et al. (2016) investigated the clinical relevance of ERO1α, particularly in triple-negative breast cancer (TNBC) [26]. Their analysis demonstrated that elevated ERO1α expression was significantly associated with reduced overall survival in breast cancer patients. These findings were consistent with previous studies, including those by Kutomi et al. [24], which linked ERO1α overexpression to more aggressive tumor characteristics. Additionally, Tanaka et al. identified ERO1α as an independent prognostic factor for survival, highlighting its potential as a biomarker in breast cancer prognosis [26].

Furthermore, Takei et al. (2019) expanded the investigation of ERO1α expression across multiple cancer types, with particular attention to breast cancer cell lines MCF-7 and MDA-MB-231 [28]. Their analysis revealed that both breast cancer cell lines exhibited elevated ERO1α protein and mRNA expression compared to normal human dermal fibroblasts (NHDF). Notably, MDA-MB-231 cells demonstrated higher ERO1α expression than MCF-7 cells, suggesting a potential association between ERO1α levels and the aggressiveness of breast cancer subtypes. The study also assessed carbonic anhydrase IX (CA9), an enzyme involved in pH regulation and cell survival commonly associated with tumor progression. Unlike CA9, which exhibited variable expression, ERO1α was consistently expressed under standard conditions.

Like Tanaka et al. [25], Varone et al. [13], examined how ERO1α expression relates to the aggressiveness in breast cancer. They analyzed various breast cancer cell lines, including MDA-MB-231, MCF-7, 4T1, CAMA1 and E0771. The highest ERO1α expression levels were observed in basal-like cell lines, particularly in triple-negative breast cancer (TNBC) cells such as MDAMB231. In contrast, luminal-type cell lines generally showed lower expression of ERO1α.

As a continuation of the study by Varone, ERO1α expression was found to be significantly higher in TNBC compared to Luminal A breast cancer. Its levels positively correlated with the proliferation marker Ki67, more strongly in TNBC, and patients with high ERO1αexpression showed a greater risk of recurrence and metastasis [33].

Recently, Hermawan et al. (2024) focused on ERO1α in cancer stem cells and its role in mammosphere formation [32]. They found that ERO1α was among the top 10 upregulated genes in MCF-7 and T47D mammospheres, suggesting that it may contribute to tumor resistance. Moreover, DNA methylation analysis showed a significantly elevated CpG methylation of ERO1α, which was associated with poor prognosis.

Interestingly, ERO1α plays a significant role in breast cancer progression, with higher expression levels linked to tumor aggressiveness, metastasis, and poor patient outcomes. Research shows that ERO1α is overexpressed in breast cancer cells and tissues, while its presence is minimal or absent in normal breast tissue. Its strong association with aggressive subtypes, such as triple-negative breast cancer, and cancer stem cell activity highlights its potential as both a prognostic marker and a therapeutic target.

### 3.2. Hypoxia Is a Major Inducer of ERO1α Expression

Hypoxia, a condition of reduced oxygen availability, is common in solid tumors due to rapid cell growth that outpace blood supply [34]. Cancer cells adapt to hypoxia by activating pathways that promote survival, growth, and metastasis [35]. Several studies have demonstrated that ERO1α plays a crucial role in the hypoxic response of cancer, particularly in regulating protein folding, angiogenesis, and adaptation to low-oxygen environments [11,23,36].

Multiple studies have found that ERO1α is highly expressed in aggressive forms of breast cancer. Varone et al. (2021) [13] reported that basal breast cancer cells, particularly TNBC cell lines, show high levels of ERO1α. In their respective studies, both Kutomi (2013) [24] and Tanaka (2015) [25] demonstrated that ERO1α levels rise under low-oxygen conditions in various cancer cell lines, including MCF-7, 4T1, and HeLa cells. Tanaka (2017) further showed that overexpression of ERO1α led to increased HIF-1α levels, suggesting that ERO1α may contribute to stabilizing HIF-1α [27]. Supporting this idea, Wang (2024) reported that silencing HIF-1α led to a decrease in ERO1α expression [31].

Beyond its effects on cell migration and tumor growth, ERO1α is also involved in protein folding and secretion under hypoxia. Varone et al. (2022) reported that cells lacking ERO1α showed defective protein folding, leading to an accumulation of misfolded proteins such as VEGF121 and the chaperone protein BIP [29], also known as GRP78, an essential ER chaperone that facilitates proper protein folding and helps manage ER stress by activating the unfolded protein response [37]. The increased levels of BIP in ERO1α-deficient cells suggest bigger ER stress under hypoxia, potentially affecting cellular function.

Interestingly, the extent of hypoxia-induced ERO1α expression appears to vary across different cancer cell lines. Varone et al. (2021) showed that while some cancer cell lines, including HeLa, MDA-MB-231, and T47D, upregulate ERO1α under hypoxic conditions, others, such as luminal CAMA1, do not show the same response [13]. The reason for this remains unclear, but it may relate to the distinct biology of CAMA1, which was derived from a pleural effusion [38] and shows evidence of CHD1 inactivation through promoter methylation [39].

This suggests that ERO1α’s regulation under hypoxia is cell-type dependent, possibly influenced by the presence of specific hypoxia-inducible factors (HIFs) or variations in the tumor microenvironment.

In summary, ERO1α is a key player in the cellular response to hypoxia, supporting protein folding, angiogenesis, and survival in low-oxygen conditions. Its expression is often elevated in aggressive breast cancer subtypes, highlighting its role in tumor adaptation to hypoxic stress. The variability in ERO1α upregulation across cell lines suggests that its response is influenced by specific hypoxia-related factors and the tumor microenvironment.

### 3.3. ERO1α Stimulates Expression of VEGF-A Leading to Increased Angiogenesis and Metastatic Potential

Angiogenesis is essential for tumor growth and metastasis, as it enables cancer cells to receive oxygen and nutrients through newly formed blood vessels [40]. VEGF-A (vascular endothelial growth factor A) is a key mediator of this process, driving blood vessel formation in response to hypoxic conditions within tumors [41]. Multiple studies have demonstrated that decreased ERO1α expression impairs angiogenesis by decreasing VEGF-A secretion, suggesting that ERO1αcan play a role in the angiogenesis process critical for cancer progression and metastasis.

Kutomi et al. (2013) reported that knocking down ERO1α in 4T1 cells significantly reduced VEGF-A secretion [24]. They proposed that hypoxia, a common feature of solid tumors, enhances VEGF-A release through ERO1α, reinforcing its role in tumor angiogenesis and metastasis.

Similarly, Tanaka et al. (2016) found that silencing of ERO1α in MDA-MB-231 cells disrupted VEGF-A secretion, which they attributed to defects in oxidative protein folding. However, VEGF-A mRNA levels did not change, indicating that ERO1α is more involved in post-transcriptional maturation, specifically through the formation of disulfide bonds, than transcriptional regulation [26]. Their study also showed that overexpressing ERO1α increased VEGF-A secretion, further supporting its role in VEGF-A processing and tumor angiogenesis.

The specific role of ERO1α in VEGF-A dimerization was highlighted by Varone et al. (2021), who found that knocking out ERO1α in MDA-MB-231 cells led to VEGF-A being secreted primarily as monomers rather than its biologically active dimer form [13]. This suggests that ERO1α is not only necessary for VEGF-A secretion but also for ensuring its correct structural configuration, which is essential for its function. They further demonstrated that reintroducing ERO1α into knockout cells restored VEGF-A dimerization and secretion, confirming its critical role in this process.

Research also consistently shows that ERO1α loss reduces VEGF-A secretion and significantly impacts angiogenesis. For example, Varone et al. (2021) observed that conditioned media from ERO1α knockout cells was less effective in promoting endothelial cell migration, a key step in angiogenesis [13]. This effect was comparable to the impact of VEGF-neutralizing antibodies, further supporting the idea that ERO1α is essential for VEGF-A processing and its role in angiogenesis. In vivo, Varone et al. (2021) found that tumors in ERO1α knockout mice grew more slowly than in wild-type mice and were more responsive to anti-VEGF-A therapy [13].

The role of ERO1α appears to become even more significant in a hypoxic tumor microenvironment. Varone et al. [30] found that under hypoxic conditions, the absence of ERO1α disrupted protein homeostasis, leading to protein aggregation and delayed VEGF-A secretion. In knockout cells, hypoxia worsened these secretion defects, highlighting the importance of ERO1α in maintaining protein stability under stress [30]. These findings align with earlier studies showing that ERO1α helps maintain redox balance in the endoplasmic reticulum, particularly under oxidative stress caused by hypoxia. Moreover, Varone et al. reported that ERO1α knockout cells had trouble secreting VEGF121, a specific VEGF variant, due to defective oxidative folding, further underscoring its role in VEGF-A maturation [30].

Taken together, these findings suggest that ERO1α is not only involved in VEGF-A secretion but also influences tumor behavior by regulating angiogenesis and metastasis. Without ERO1α, VEGF-A maturation is disrupted, reducing its ability to promote angiogenesis, leading to slower tumor growth and decreased metastatic potential.

### 3.4. ERO1α Mediates mTORC1 Activated Ferroptosis Resistance

Ferroptosis is a form of regulated cell death driven by iron-dependent lipid peroxidation, distinct from apoptosis or necrosis [42]. It plays a complex role in cancer, acting as both a tumor suppressor and a potential vulnerability point for aggressive cancers [43]. While some tumors develop resistance by enhancing antioxidant defenses, particularly through the SLC7A11-GPX4 pathway, others remain highly sensitive to ferroptotic cell death [44]. For example, the simultaneous upregulation of GPX4 and ACSL3 prevents lipid peroxidation and protects pancreatic cancer cells from ferroptosis both in vitro and in vivo, as shown through IL15RA-STAT3-GPX4/ACSL3 signaling [45]. In contrast, loss of GPX4 function results in selective ferroptotic death of drug-tolerant persister cells and prevents tumor relapse in vivo, as demonstrated in breast cancer, non-small cell lung cancer, and ovarian cancer models [46].

Wang (2024) investigated how ERO1α affects ferroptosis resistance, particularly in mTORC1-activated cancer cells [31]. Using Tsc1- and Tsc2-null mouse embryonic fibroblasts (MEFs), authors showed that ERO1α levels increased with mTORC1 activation and were reversed by rapamycin. Functionally, ERO1α promoted proliferation, angiogenesis, and tumor growth in Tsc2-deficient cells, both in vitro and in vivo. Additionally, ERO1α contributed to ferroptosis resistance by upregulating SLC7A11, maintaining redox balance, and protecting mitochondrial integrity. In addition, knockout of ERO1α sensitized cells to ferroptosis and reduced tumor growth.

This regulatory mechanism was further validated in human cancer cells by Wang et al. [31] in the same study. In LSCC models, inhibition of mTORC1 suppressed the expression of ERO1α, IL-6, p-STAT3, and SLC7A11, whereas TSC2 knockout enhanced their levels. Importantly, Wang also reported that genetic or pharmacological inhibition of mTORC1 led to a similar suppression of this signaling network in several mTORC1-hyperactivated cancer cell lines, including the triple-negative breast cancer cell line MDA-MB-231 [31]. This finding confirms that the mTORC1/ERO1α/IL-6/STAT3/SLC7A11 axis is conserved across cancers and has direct relevance to breast cancer progression.

These findings show that ERO1α plays an important role in helping cancer cells resist ferroptosis through the mTORC1/IL-6/STAT3/SLC7A11 signaling pathway. By maintaining redox balance and supporting mitochondrial function, ERO1α helps cancer cells survive and grow, especially in tumors with high mTORC1 activity. However, the exact ways ERO1α affects ferroptosis are still not fully clear. More research is needed to better understand this process and explore whether targeting ERO1α could be a useful strategy for cancer treatment.

### 3.5. ERO1α Expression Modulates Immune Response and Tumor Microenvironment

#### 3.5.1. By Inhibiting T-Cell Response via Recruitment of Myeloid-Derived Suppressor Cells (MDSCs)

Tumor microenvironment is a complex and dynamic environment surrounding the tumor, which consists of a multitude of different components such as malignant cells, non-cancerous cells, immune cells, extracellular matrix components, blood vessels, and others. Tumor microenvironment has been shown to play a significant role in cancer development and progression as well as in sensitivity to anti-cancer therapy. Often, tumor microenvironment has immunosuppressive character. Malignant cells constantly adapt to changing microenvironment and have evolved mechanisms to evade immune surveillance system of the host. This is often achieved by recruitment and accumulation of immunosuppressive cells such as myeloid-derived suppressor cells (MDSCs), regulatory T cells, tumor-associated macrophages, and others [47]. The role of MDSC cells in cancer is increasingly recognized due to their ability to modulate anti-tumor immunity via various mechanisms. MDSC cells can both inhibit immune-active cells such as effector T cells and natural killer cells and stimulate other inhibitory immune cells such as regulatory T cells and regulatory B cells [48]. There are two main types of MDSC cells: polymorphonuclear MDSC cells (PMN-MDSC) and monocytic MDSC cells (M-MDSC) [49].

Tanaka et al. [25] have shown that ERO1α expression markedly inhibits T-cell mediated anti-tumor immunity in 4T1 mouse breast cancer cells. On the contrary, knock-down of ERO1α results in enhanced anti-tumor immunity [25]. Authors have also demonstrated that mice with overexpression of ERO1α had higher levels of PMN-MDSCs infiltration in spleen, bone marrow, peripheral blood and tumor compared to mice bearing mock tumors. These results suggest that ERO1α expression within the tumor leads to recruitment and accumulation of PMN-MDSCs, which can be responsible for inhibition of T-cell-mediated anti-tumor immunity. This was further supported by the fact that depletion of tumor-associated PMN-MDSCs in ERO1α+ 4T1 cells inhibited tumor growth compared to control.

Moreover, authors have also investigated mechanisms of how ERO1α induces accumulation of PMN-MDSCs within the tumor. They found that ERO1α+ cells produce higher levels of granulocyte colony-stimulating factor (G-CSF) compared to ERO1α knockdown cells. G-CSF have been previously shown to play an important role in induction and proliferation of PMN-MDSC cells [50]. Therefore, ERO1α have a positive regulatory effect on G-CSF, which in turn is responsible for proliferation of PMN-MDSC cells. Moreover, besides G-CSF there are other cytokines such as CXCL1 and CXCL2, which are also known for their ability to recruit PMN-MDSC cells from the circulation into the tumor stroma [50]. Tanaka et al. [25] demonstrated not only increased levels of CXCL1 and CXCL2 in supernatant of ERO1α+ 4T1 cells compared to ERO1A knockdown cells but also increased expression of CXC receptors (CXCR2) on PMN-MDSC cells in spleen, bone marrow and peripheral blood. Therefore, ERO1α+ tumor cells secrete G-CSF, which acts as a proliferation factor for PMN-MDSC cells as well as CXCL1 and CXCL2, which stimulate PMN-MDSC cells recruitment from the circulation into the tumor stroma.

Interestingly, ERO1α affected protein expression levels of G-CSF, CXCL1 and CXCL2 but not mRNA expression. Authors have demonstrated that ERO1α promotes oxidative protein folding of G-CSF, CXCL1 and CXCL2 proteins; hence, facilitating their production.

Overall, these findings demonstrate that ERO1α expression promotes tumor-suppressive microenvironment via recruitment and accumulation of PMN-MDSC cells mainly by facilitating proper protein folding of cytokines needed for recruitment of PMN-MDSC cells such as G-CSF, CXCL1 and CXCL2.

#### 3.5.2. By Stimulating Expression of PD-L1 and Decreasing Anti-Tumor Immunity

Another mechanism on how ERO1α expression can modulate anti-tumor immunity is via upregulation of Programmed Cell Death Ligand 1 (PD-L1). PD-L1 and its receptor Programmed Cell Death Protein 1 (PD-1) belong to immune checkpoint proteins due to their role in preventing undesirable autoimmune responses in physiological conditions [51]. Cancer cells, however, have exploited PD-L1/PD-1 signaling pathway to impede anti-tumor immunity via inhibition of T-cell activity [52]. Like PMN-MDSC cells, PD-L1 also plays a significant role in promoting immunosuppressive tumor microenvironment.

PD-1 is normally expressed on various immune cells such as B-cells, T-cells, and natural killer cells [51]. PD-L1, in turn, is overexpressed in several cancer types, including breast cancer, and its overexpression correlates with poor cancer prognosis [53]. Activation of PD-1 by PD-L1 secreted from cancer cells inhibits T-cell activity, disrupting tumor immune surveillance and promoting tumor progression. Thus, targeting PD-L1/PD-1 signaling pathway has become an important target in cancer immunotherapy.

A study by Tanaka et al. [27] highlighted the role of ERO1α in regulating PD-L1 expression in TNBC. The researchers found that ERO1α enhances PD-L1 expression through two mechanisms: by facilitating oxidative protein folding and increasing transcription via HIF-1α [27].

When ERO1α was overexpressed in MDA-MB-231 TNBC cells, there was a substantial increase in PD-L1 surface levels and PD-L1 mRNA expression. This upregulation of transcription was linked to the stabilization of HIF-1α, which was driven by the ROS accumulation resulting from ERO1α activity. Conversely, silencing ERO1α led to a significant reduction in PD-L1 surface expression without affecting PD-L1 mRNA levels, indicating that ERO1α is essential for the oxidative maturation of the PD-L1 protein [27].

**Figure 2:**
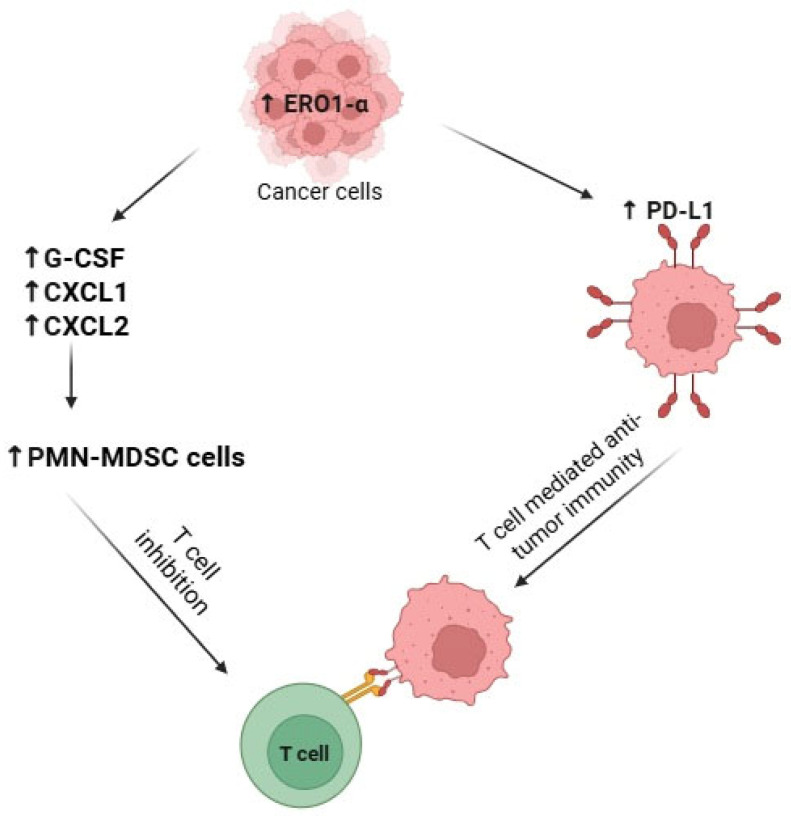
ERO1α-mediated immunosuppressive mechanism in the tumor microenvironment. Upregulation of ERO1α in cancer cells enhances the secretion of pro-inflammatory cytokines and chemokines such as G-CS), CXCL1, and CXCL2, which promote the recruitment and expansion of polymorphonuclear myeloid-derived suppressor cells (PMN-MDSCs). Simultaneously, ERO1α upregulates PD-L1 expression, further suppressing T cell-mediated anti-tumor immunity.

Further proof that ERO1α is responsible for the maturation of PD-L1 protein came from the experiment with ERO1α inhibitor EN460, which is known to inhibit oxidative protein folding via reductive inactivation of ERO1α. Treatment of MDA-MB-231 cell line with EN460 resulted in decreased expression of PD-L1 protein levels compared to non-treated control, further highlighting the role of ERO1α in correct folding of PD-L1 protein. Moreover, to demonstrate the consequences of ERO1α-mediated overexpression of PD-L1 for anti-tumor immunity, authors showed that coculture of Jurkat leukemia T cells with ERO1α-expressing cells resulted in enhanced apoptosis of T cells compared to ERO1α knock-down cells. Overall, these results demonstrate that ERO1α overexpression can confer immunosuppressive phenotype due to inhibition of T cell activity via upregulation of PD-L1 protein levels. These two pathways are summarized in Figure 2.

Altogether, these results highlight the importance of ERO1α in facilitating anti-tumor immunity mainly via two mechanisms: recruitment of immunosuppressive PMN-MDSC cells and increased production of immune checkpoint protein PD-L1. Ultimately these two different mechanisms work together to inhibit T-cell mediated anti-tumor immunity and help cancer cells to evade the immune system of the host.

## 4. Discussion

This review offers an in-depth analysis of the multifactorial role of ERO1α in breast cancer development and progression. Beyond its well-characterized function in oxidative protein folding, ERO1α contributes to several tumor-promoting processes, including hypoxia adaptation, ER stress mitigation, and angiogenesis through proper VEGF-A maturation. Its involvement in sustaining cancer stem cell properties, modulating immune responses through cytokine secretion and PD-L1 expression, and enhancing resistance to ferroptosis further illustrates its central role in maintaining an aggressive tumor phenotype.

Overexpression of ERO1α is widely reported across various cancer types and often associated with poor clinical outcomes, highlighting its role in tumor survival. For example, elevated levels of ERO1α in lung, esophageal hepatocellular carcinoma and diffuse B-cell lymphoma, elevated ERO1α levels have been associated with bad prognosis [15]. This general oncogenic role of ERO1α aligns closely with our review’s findings in breast cancer, where consistent evidence shows markedly higher ERO1α expression in breast cancer cells compared to normal mammary tissue [24,25]. Particularly, the preferential expression in aggressive basal-like and TNBC subtypes mirrors the pattern observed in other highly invasive cancers, reinforcing its association with tumor aggressiveness [13].

Emerging evidence indicates that ERO1α contributes to cancer stem cell maintenance and therapy resistance, and its regulation through DNA methylation provides a mechanistic link consistent with observations across diverse tumor contexts. Although luminal breast cancer cell lines generally display low basal levels of ERO1α, their ability to form mammospheres enriches for a stem-like population in which ERO1α is among the most strongly upregulated genes, as demonstrated in MCF-7 and T47D models [32]. This suggests that ERO1α expression is inducible in primitive luminal cancer stem-like cells, thereby integrating stemness, epigenetic control, and tumor adaptation. Collectively, these findings support the notion that ERO1α acts as a key driver of cancer progression and represents a promising therapeutic target across multiple cancer types, including breast cancer.

Hypoxia is a well-established and consistent hallmark of solid tumors and plays a pivotal role in shaping the tumor microenvironment by activating adaptive survival mechanisms [54]. The findings of the current analysis showed ERO1α as a key mediator of cellular adaptation to hypoxic stress. Broadly across cancer types, hypoxia induces ERO1α expression through the activation of HIFs, particularly HIF-1α, which transcriptionally upregulates ERO1α [28]. Intriguingly, ERO1α itself has been shown to stabilize HIF-1α, forming a feed-forward loop that enhances cellular resilience under low-oxygen conditions [26]. This dual relationship underscores ERO1α’s critical role in supporting tumor cell survival, angiogenesis, and metabolic adaptation. For breast cancer, the findings in this review clearly align with this broader pattern, reinforcing the idea that ERO1α is hypoxia-responsive but also revealing important subtype-specific nuances. Furthermore, by maintaining ER homeostasis, ERO1α allows cancer cells to continue secreting pro-tumorigenic factors and avoid activation of the UPR [30].

An important functional role of ERO1α in cancer biology lies in its role in promoting angiogenesis, primarily through the post-transcriptional regulation of VEGF-A [26]. As a crucial oxidative folding enzyme within the endoplasmic reticulum, ERO1α ensures the correct disulfide bond formation required for VEGF-A dimerization and secretion. Disruption or knockdown of ERO1α leads to improperly folded VEGF-A, reduced secretion, and consequently, impaired angiogenesis, which translates to delayed tumor growth and limited vascular development [55]. This role is especially pronounced under hypoxic conditions, where tumors rely heavily on VEGF-A-driven neovascularization to overcome oxygen and nutrient limitations [56]. Our review findings in breast cancer are consistent with this mechanism, further emphasizing that ERO1α supports VEGF-A activity not at the level of gene transcription, but through essential post-translational quality control.

Recent studies have expanded our understanding of ERO1α’s oncogenic role, linking it to pathways beyond oxidative protein folding. A notable example is its regulation of ferroptosis resistance through the mTORC1/IL-6/STAT3/SLC7A11 axis. Ferroptosis, an iron-dependent form of regulated cell death, is increasingly recognized as a vulnerability in apoptosis-resistant cancers [43]. ERO1α appears to promote ferroptosis resistance by sustaining redox balance, preserving mitochondrial integrity, and activating antioxidant systems via mTORC1 signaling [31]. Although evidence directly connecting ERO1α to ferroptosis remains limited, Wang et al. [57] demonstrated that in cholangiocarcinoma (CCA) cells, 2′,4′-dihydroxychalcone (2′,4′-DHC) targets ERO1α, inducing GPX4 degradation and ferroptosis. In vivo, 2′,4′-DHC suppressed tumor growth and enhanced oxaliplatin efficacy without systemic toxicity, highlighting the translational promise of combining ERO1α inhibition with ferroptosis inducers and chemotherapy [57]. These findings suggest that co-targeting ERO1α and ferroptotic pathways may offer a novel therapeutic strategy, particularly for therapy-resistant breast cancer, though systematic validation in preclinical models is still required.

In parallel, ERO1α has also been shown to modulate the tumor immune microenvironment—specifically, it enhances the oxidative folding and secretion of immunosuppressive cytokines such as G-CSF, CXCL1, and CXCL2, which are instrumental in recruiting PMN-MDSCs [25]. These cells inhibit T-cell-mediated anti-tumor responses, facilitating immune escape and supporting tumor growth [58]. Furthermore, recent evidence suggests that ERO1α upregulation may contribute to increased PD-L1 expression on tumor cells, further dampening T-cell activity and reinforcing immune evasion mechanisms [27]. This immunomodulatory role of ERO1α positions it as a central mediator of tumor immune evasion. Together, these mechanistic insights emphasize that ERO1α contributes not only to tumor cell survival and angiogenesis but also to resistance against ferroptosis and immune attack. As such, ERO1α represents a promising target for combinatorial therapies aimed at enhancing ferroptosis sensitivity and reversing tumor immune suppression.

Despite growing recognition of its role in tumor biology, research on ERO1α remains limited, with only a small number of studies—11 in breast cancer to date—directly investigating its functions, mechanisms, and therapeutic potential. ERO1α was first reported and characterized in mammals in 2000, sharing extensive homology with the Saccharomyces cerevisiae ERO1 gene and establishing its role in oxidative protein folding within the endoplasmic reticulum [59]. Compared to well-established biomarkers, this makes ERO1α a relatively new target in oncology, with its precise contribution to tumor progression and therapy resistance still underexplored. ERO1α functions in close cooperation with PDI, and while both proteins are central to disulfide bond formation and redox regulation, it remains unclear which exerts predominant control in this pathway. Given that both are overexpressed in tumors, a critical question is whether inhibition of ERO1α acts in part by modulating PDI activity, and how this differs mechanistically from directly targeting PDI [16].

Notably, despite their biological importance, no clinically approved inhibitors exist for either ERO1α or PDI. The most studied small molecule ERO1α inhibitor, EN460, contains a Michael acceptor moiety that forms a covalent adduct with an essential cysteine residue, thereby trapping ERO1α in a reduced state and preventing electron transfer to PDI. While EN460 shows target engagement in vivo, its conjugated double bond raises concerns about off-target reactivity with thiol-containing compounds, limiting its clinical applicability [22]. Cell-based and in vivo screening campaigns have since yielded promising derivatives, such as I2 and I3, which inhibit VEGFA secretion by trapping ERO1α in its reduced inactive form. I2 demonstrated superior activity to EN460 in triple-negative breast cancer models by reducing VEGF secretion and PD-L1 expression, while showing no apparent systemic toxicity in vivo [33]. However, even these newer compounds retain partial thiol reactivity in vitro, highlighting the persistent challenges of achieving both potency and specificity. Collectively, these findings emphasize that while ERO1α represents an attractive therapeutic target, substantial gaps remain, particularly the lack of clinical validation, the absence of standardized measurement methods, and the need for clinically viable inhibitors that can reliably distinguish between ERO1α- and PDI-directed effects.

Despite these significant findings, several limitations should be noted. First, most available studies are preclinical and rely on in vitro cell line models or xenograft systems, which do not fully capture the complexity and heterogeneity of human breast tumors. Direct comparisons are further complicated by substantial variation in study design, experimental conditions, and outcome measurements. For example, while luminal-type cell lines such as MCF-7 and T47D consistently upregulate ERO1α under hypoxic conditions, other luminal lines like CAMA-1 do not, highlighting context-dependent responses that remain poorly understood. These inconsistencies restrict the generalizability of current findings and underscore the need for systematic cross-line investigations.

Publication bias is another possibility, as no systematic search was performed for unpublished data or conference abstracts. Furthermore, given the rapidly evolving nature of the field, relevant studies published after the final search date may not have been captured.

Extending the clinical validation of ERO1α as a predictive biomarker in breast cancer should be the primary focus of future research. This should be accomplished primarily through large-scale cohort studies that incorporate molecular profiling with patient outcomes. To better understand its function in therapy resistance, mechanistic investigations are also required to analyze the interplay between ERO1α and other stress-adaptive pathways such as autophagy, ferroptosis management, and immunological checkpoint signaling. Furthermore, because of its several tumor-promoting roles, it is crucial to prioritize the development of selective and potent ERO1α inhibitors.

## 5. Conclusions

In summary, this systematic review highlights ERO1α as a critical facilitator of multiple oncogenic processes in breast cancer, particularly within basal-like and triple-negative subtypes. Rather than acting through a single dominant pathway, ERO1α influences a range of interconnected mechanisms that support tumor survival, immune evasion, and treatment resistance. These findings position ERO1α as a compelling candidate for targeted therapies and suggest that its inhibition could enhance the efficacy of existing treatments by disrupting key adaptive responses in cancer cells.

## Figures and Tables

**Table 1:** Summary of the studies included in the review.

#	Year	Authors	Name	Species	Cell Lines	Key Findings
1	2013	Kutomi et al. [24]	Human endoplasmic reticulum oxidoreductin 1-α is a novel predictor for poor prognosis of breast cancer	Human, mice	4T1	ERO1α is overexpressed in breast cancer and promotes tumor progression and metastasis. Its mRNA is detected in breast cancer tissues but not in normal tissues, and its overexpression is confirmed in MCF7 cells. Knockdown of ERO1α in 4T1 cells leads to reduced tumor growth, fewer lung metastases, and decreased VEGF-A production.
2	2015	Tanaka et al. [25]	Cancer-associated oxidoreductase ERO1-α drives the production of tumor-promoting myeloid-derived suppressor cells via oxidative protein folding	Human, mice	4T1 MCF7 BT-474 UACC-893 SK-BR-3 MDA-MB-15 MDA-MB-231 and MDA-MB-468	ERO1α promotes tumor growth and immune evasion in breast cancer by enhancing the secretion of immunosuppressive cytokines. Knockdown of ERO1α in 4T1 cells reduced tumor growth and improved survival in immunocompetent mice, effects that were lost when CD4^+^ and CD8^+^ T cells were depleted, highlighting its role in suppressing T cell-mediated immunity. ERO1α-overexpressing tumors had higher levels of G-CSF, CXCL1, and CXCL2 proteins, leading to PMN-MDSC accumulation and immune suppression, despite unchanged mRNA levels.
3	2016	Tanaka et al. [26]	Cancer-associated oxidoreductase ERO1-α drives the production of VEGF via oxidative protein folding and regulating the mRNA level	human, mice	MDA-MB-157 MDA-MB-231 MDA-MB-468 MCF7	ERO1α is significantly upregulated in TNBC cell lines and tissues, correlating with poorer overall survival in patients. Knockdown of ERO1α in MDA-MB-231 cells slowed tumor growth and reduced tumor angiogenesis, as shown by fewer CD31+ blood vessels, while overexpression led to more aggressive tumor growth in NOD/SCID mice. ERO1α regulates VEGF at the protein level without altering its mRNA expression—knockdown decreased the mature, oxidized form of VEGF, and inhibition with EN460 reduced VEGF protein secretion. Additionally, ERO1α overexpression increased HIF-1α and reactive oxygen species.
4	2017	Tanaka et al. [27]	Cancer-associated oxidoreductase ERO1-α promotes immune escape through up-regulation of PD-L1 in human breast cancer	human, mice	MDA-MB-231 MDA-MB-468	ERO1α enhances PD-L1 expression and maturation in MDA-MB-231 cancer cells. OE of ERO1-α increased both PD-L1 surface protein and mRNA levels, while KD reduced them. OE cells showed higher HIF-1α protein and ROS levels, and silencing HIF-1α lowered PD-L1 mRNA in some cells. ERO1α promoted the oxidized (mature) form of PD-L1, with KD cells showing a significantly lower oxidized-to-reduced PD-L1 ratio.
5	2019	Takei et al. [28]	ERO1α is a novel endogenous marker of hypoxia in human cancer cell lines	human, mice	MDA-MB-231 MCF7	Under normoxic conditions, ERO1α is expressed across all tested cell lines, with notably higher levels in cancer cell lines. Additionally, the hypoxia marker CA9 is significantly elevated in the aggressive MDA-MB-231 breast cancer cells compared to both normal cells and MCF7 cells.
6	2021	Varone et al. [13]	The ER Stress Response Mediator ERO1 Triggers Cancer Metastasis by Favoring the Angiogenic Switch in Hypoxic Conditions	Human, mice	MDAMB231 4T1 E0771	ERO1α is highly expressed in several breast cancer cells, particularly elevated in aggressive basal/TNBC types. Under hypoxia, ERO1α levels increase in most cells except luminal CAMA1. Loss of ERO1α impairs cell migration and leads to an accumulation of proteins with free thiols and reduced disulfide-bonded secreted factors. VEGFA secretion is significantly decreased in ERO1α KO cells, especially under hypoxia, while VEGFR2 is upregulated, possibly as compensation. Key ER stress markers ATF4 and CHOP are downregulated in ERO1α KO cells during hypoxia, unlike in wild-type cells where they increase, indicating impaired unfolded protein response activation.
7	2022	Varone et al. [29]	Endoplasmic reticulum oxidoreductin 1-alpha deficiency and activation of protein translation synergistically impair breast tumour resilience	Human, mice	MDAMB231 MCF7	Under hypoxia, ERO1α KO cells showed increased accumulation of VEGF121 and chaperone BIP in the detergent-insoluble fraction, along with higher phosphorylated eIF2α, indicating suppressed protein translation. While wild-type MDA-MB-231 cells maintained protein synthesis under hypoxia, ERO1α KO cells exhibited reduced translation. ISRIB modestly decreased ATF4 and CHOP transcripts without affecting ERO1α expression. VEGFA expression was reduced in ERO1α KO cells under hypoxia, whereas VEGFB remained unchanged. Additionally, ERO1α KO breast tumors upregulated the PERK pathway of the unfolded protein response.
8	2022	Varone et al. [30]	ERO1 alpha deficiency impairs angiogenesis by increasing N-glycosylation of a proangiogenic VEGFA	mice	ERO1 KO-MDAMB231	ERO1α KO TNBC xenografts exhibited significantly increased protein N-hyperglycosylation compared to wild-type tumors, with a five-fold increase in cluster volume observed in ERO1α KO tumors.
9	2024	Wang et al. [31]	Augmented ERO1α upon mTORC1 activation induces ferroptosis resistance and tumor progression via upregulation of SLC7A11	Human, mice	MDA-MB-231	ERO1α acts as a downstream effector of mTORC1, promoting ferroptosis resistance and tumor progression by upregulating SLC7A11 through activation of the IL-6/STAT3 pathway. Combining ERO1α inhibition with the ferroptosis inducer imidazole ketone erastin (IKE) produced a synergistic antitumor effect in mTORC1-driven tumor models, including cell line xenografts, LSCC organoids, and patient-derived xenografts.
10	2024	Hermawan et al. [32]	Transcriptomics analysis reveals distinct mechanism of breast cancer stem cells regulation in mammospheres from MCF-7 and T47D cells	human	MCF-7 T47D cells	ERO1α ranked among the top 10 upregulated genes in breast cancer. Additionally, DNA methylation analysis showed significant differences in ERO1L gene expression between low-risk and high-risk breast cancer patient groups.
11	2025	Varone et al. [33]	Small molecule-mediated inhibition of the oxidoreductase ERO1A restrains aggressive breast cancer by impairing VEGF and PD-L1 in the tumor microenvironment	Human, mice	MDA-MB-231	ERO1A is overexpressed in MDA-MB-231 cell line and drives breast cancer aggressiveness. EN460 and I2 downregulated proliferative pathways (E2F, G2M, MYC), consistent with suppressed tumor growth. ERO1A inhibition limits tumor fitness by impairing proliferation, suppressing angiogenesis, and modulating the immune microenvironment.

## Abbreviations

The following abbreviations are used in this manuscript:

BIP/GRP78	Binding Immunoglobulin Protein/Glucose-Regulated Protein 78
Breg	Regulatory B Cell
CpG	Cytosine-phosphate-Guanine
CXCL1/CXCL2	C-X-C Motif Chemokine Ligand 1/2
CXCR2	C-X-C Chemokine Receptor Type 2
CCA	Cholangiocarcinoma
ER	Endoplasmic Reticulum
ERO1α	Endoplasmic Reticulum Oxidoreductin 1 Alpha
G-CSF	Granulocyte Colony-Stimulating Factor
GPX4	Glutathione Peroxidase 4
HIF-1α	Hypoxia-Inducible Factor 1 Alpha
MDSC	Myeloid-Derived Suppressor Cell
M-MDSC	Monocytic Myeloid-Derived Suppressor Cell
mRNA	Messenger Ribonucleic Acid
mTORC1	Mechanistic Target of Rapamycin Complex 1
NK Cell	Natural Killer Cell
PDI	Protein Disulfide Isomerase
PD-1	Programmed Cell Death Protein 1
PD-L1	Programmed Death-Ligand 1
PMN-MDSC	Polymorphonuclear Myeloid-Derived Suppressor Cell
PRISMA	Preferred Reporting Items for Systematic Reviews and Meta-Analyses
ROS	Reactive Oxygen Species
SLC7A11	Solute Carrier Family 7 Member 11
TME	Tumor Microenvironment
Treg	Regulatory T Cell
TSC1/TSC2	Tuberous Sclerosis Complex 1/2
UPR	Unfolded Protein Response
VEGF-A	Vascular Endothelial Growth Factor A
VEGF121	Vascular Endothelial Growth Factor 121 Isoform
GRADE	Grading of Recommendations Assessment, Development and Evaluation
